# Future-minded: the role of prospection in Agency, Control, and other goal-directed processes

**DOI:** 10.3389/fpsyg.2015.00154

**Published:** 2015-02-17

**Authors:** Magda Osman

**Affiliations:** Biology and Experimental Psychology Centre, Queen Mary University of LondonLondon, UK

**Keywords:** prospection, feedforward, control, planning, memory

## What is so important about prospection?

Placing the future center stage as a way of understanding the function of various cognitive processes is a theoretical position that is gaining in interest and its origins can be traced to work on foresight in the 60s (Stark, 1966) and 70s (Fischhoff, 1975). In fact, some might say that prospection was at the heart of cybernetics, and so its origins lie in work dating back to the 40s (Wiener, 1948). This work described systems (e.g., biological systems, ecologies, societies, economies, industrial power plants, banking systems, transport networks) that operate according to feedback and feed-forward loops. Consider the analogy of our circulatory system in which blood is the vehicle for carrying oxygen around the body. Essentially, the claim cyberneticists were making was that any self-regulating system involves information traveling through it in a manner that helps to reach and maintain the goals of the system (e.g., homeostasis). To achieve this, the system has to be flexible enough to enable some adjustment because information being repeatedly fedback through the system has to reflect adaptation and change (e.g., the organism running and therefore requiring more oxygen than when it is in a restful state). A self-regulating system is also anticipatory, this is because it has to be prepared for future changes in state and different demands placed on it, and to achieve this information fedback through the system is used to make predictions (feed-forward). Therefore, two complementary ways in which information is used to help a system self-regulate efficiently is to accurately relaying information back into the system in order for learning to occur, and accurately use the information relayed in order to adapt to the future. The same concepts fundamentally apply to cognition (human and animal), because any learning system requires feedback and feedforward processes, and so prospection, is embedded in all aspects of our behavior because we learn to adapt to change, and we can't do that without anticipation of the future (Osman, 2010, 2014a).

## What does prospection mean within the psychology community?

The aim of this special issue was to bring the research community's attention to an area of research in psychological science that is gaining momentum, and that offers a way of understanding human behavior from the point of future-oriented cognition.

In this special issue titled “Future-minded: the role of prospection in Agency, Control, and other goal-directed processes” there is a range of contributions that include empirical studies examining prospection and emotional memories in a sleep paradigm (Cunningham et al., 2014), the connection between prospective memory and emotions in decision-making (Worthy et al., 2014), and the connection between prospection in the context of deception (Knieps et al., 2014). There are also three review papers that focus on different aspects of prospection such as the broad conceptual issues around prospection (Osman, 2014b), the current insights from work on prospective memory (Walter and Meier, 2014), and the links between prospective memory and imagination (Zheng et al., 2014).

Unlike many specially issues, all the contributors (Cunningham et al., 2014; Knieps et al., 2014; Walter and Meier, 2014; Worthy et al., 2014; Zheng et al., 2014) took the time to define the concept that they were examining. This provides an important insight into the way in which this emerging field of research is conceptualized by the various researchers that are interested in it. Worthy et al. (2014) treats prospection as “… the representation and consideration of the future value of decisions” and Zheng et al. (2014) claim that “… it allows individuals to ensure their future interest and prevent future losses in advance” consider the role of values and outcomes as a key factor in prospection. Knieps et al. (2014) proposes that prospection involves “… thinking about possible future states of the world” and Osman (2014b) claims that it is “the process of representing and planning for possible future states of the world,” both electing for a much broader conceptualization of the label prospection.

One area of research in which prospection has gained most attention has been in memory, and here we have two complementary definitions from the contributors to the special issue, Walter and Meier (2014) suggest that prospective memory is “… the ability to plan, retain and retrieve an intention as planned.” and Cunningham et al. (2014) claim that it “… involves the intention formation of a memory so that information can be recalled and acted upon at a later time.”

Clearly, in their definitions, the common theme amongst the contributors is that prospection is goal-directed, and the goal is to coordinate cognitive processes, actions, or sequences of behaviors that are designed to meet a possible future demand on the agent (responsive-mode), or work toward achieving a desirable outcome for the agent, or a group (proactive-mode). Is prospection a type of meta-process that coordinates a variety of cognitive processes in a goal-specific way? Or is it essentially a representational feature of all cognitive processes, in that it can be characterized as a type of framework for encoding and adapting memories in order to represent future state of the world in each aspect of cognition (e.g., memory, attention, perception, decision-making, reasoning, imagination)? In other words, is prospection better thought of as a process, or as a type of representation, or should we think of it as a combination of both?

Based on the contributions to this special issue, Osman (2014b), Worthy et al. (2014), Zheng et al. (2014) treat prospection as a process that concerns future outcomes, whereas Cunningham et al. (2014) and Knieps et al. (2014) refer to prospection as if it is a type of representation of future states that in turn effects processes, and Walter and Meier's (2014) review strongly implies that prospection both coordinates various processes through intentions, and also influences our representational system so that we can generate imagined future states of the world. All of these different views compliment each other, but as yet, we are not quite at the stage where there is a single computational framework that underpins all of these different aspects of prospection. That is to come.

### Conflict of interest statement

The author declares that the research was conducted in the absence of any commercial or financial relationships that could be construed as a potential conflict of interest.
